# Supplementary material for: Structural dynamics of DNA strand break sensing by PARP-1 at a single-molecule level
Source: Nat Commun. 2022 Nov 2;13:6569. doi: 10.1038/s41467-022-34148-1 (PMC9630430; doi:10.1038/s41467-022-34148-1)
Supplement: Supplementary file 1 — Supplementary Information [file 41467_2022_34148_MOESM1_ESM.pdf]

Supplementary information

Structural dynamics of DNA strand break sensing by PARP-1 at a single-molecule level

Anna Sefer[1]*, Eleni Kallis[1]*, Tobias Eilert[2], Carlheinz Röcker[1], Olga Kolesnikova[3] David Neuhaus[4], Sebastian Eustermann[3†], Jens Michaelis[1†]

[1] Institute of Biophysics, Ulm University, Albert-Einstein-Allee 11, 89081 Ulm, Germany

[2]Boehringer Ingelheim, CoC CMC Statistics & Data Science, Birkendorfer Str. 65, 88400 Biberach

[3]European Molecular Biology Laboratory (EMBL), Heidelberg Meyerhofstraße 1, 69117, Heidelberg, Germany.

[4] MRC Laboratory of Molecular Biology, Francis Crick Avenue, Cambridge Biomedical Campus, Cambridge, CB2 0QH, U.K.

Supplementary Methods

Gel electrophoresis

The ligation efficiency was assessed via gel electrophoresis. Phosphorylated DNA, ligated DNA and the control lacking the ligase were loaded on a 15% PAA 1xTBE 7M urea gel (16x20cm). The gel was prerun for 1.5hours at 300 V and run for 8.5hours. The gel was imaged on a ChemiDoc MP imaging system (BioRad)

Electromobility shift assays.

Band shift assays were carried out using 6% polyacrylamide gel as described previously [1]with minor modifications. 0.5xTB buffer with 5% glycerol was used for gel preparation and as running buffer. Gels were prerun at 55V for 20 min at 4C. 50nm of DNA_{Tamra} ligand (methods) was mixed with protein in binding buffer (20mM Tris-HCl, pH 7.5, 100 mM NaCl, 3mM MgCl2, 150 µM ZnSO4, 4mM DTT, 10% glycerol) in a total volume of 10µL. Prior to gel electrophoresis, samples were incubated for 30 min at room temperature. Gel electrophoresis was conducted for 50 min at 55V at 4°C. Fluorescent DNA signal was detected using Typhoon FLA 9500 instrument (GE Healthcare).

Determination of the isotropic Förster radius

In order to quantify the isotropic Förster radius, several parameters had to be measured for the dyes in context of the DNA construct. The quantum yield of the donor attached to the DNA in absence of the acceptor was determined by product of the lifetime (Supplementary methods) and the radiative rate constant. The lifetime of the donor in absence of the acceptor was obtained from the subensemble analysis of the donor-only population of the smFRET measurement of donor only strand for $DNA_{Atto550}$ (methods) and from smFRET measurement of DNA_{Tamra}. For the radiative rate constant of Atto550 the value given by the manufacturer was used. As for 6-Tamra, the radiative rate constant was quantified by measuring the lifetime and quantum yield of unconjugated 6-Tamra. To calculate the spectral overlap *J* for $DNA_{Atto550}$ the emission of the donor strand was recorded. For the alternative DNA construct, DNA_{Tamra}, to measure the emission spectrum of the donor in absence of FRET, a reference sample (double stranded DNA, internally labeled with 6-Tamra and diluted in the buffer used for smFRET measurements) had to be used. Both donor emission spectra were recorded on a SPEX Fluorolog II (Horiba) (spectral bandwidth of 4.25nm for the excitation and 2.13nm for the emission) under magic angle conditions. For measuring the absorption spectrum of the acceptor, a DNA strand comprising only the acceptor labeled 3' stem of the DNA_{AT550} (see section on synthesis of the DNA ligand) was diluted in the buffer also used for smFRET measurements. The spectrum was taken on a Cary 50 Bio spectrophotometer (Varian). Both spectra are

needed to determine the overlap integral. Additionally, the absorption coefficient of Alexa647 at the absorption maximum (651nm determined from the measured spectrum) is needed and given as 270000 $M^{-1}cm^{-1}$ by the manufacturer (ThermoFisher Scientific). A value of 1.35 was assumed for the refractive index of the medium between the two dyes. PhotochemCAD 3 [2] was used to calculate the isotropic Förster distance R_{iso}=70Å for DNA_{Atto} R_{iso}=68Å for DNA_{Tamra} [3] from the determined parameters.

The uncertainty for the isotropic Förster radius ΔR_{iso} was determined according to equation 1 by error propagation of errors in the refractive index *n*, the donor quantum yield ϕ and the overlap integral *J*.

$$\Delta R_{iso} = \sqrt{\left(\Delta R_{iso}(n)\right)^2 + \left(\Delta R_{iso}(\Phi)\right)^2 + \left(\Delta R_{iso}(J)\right)^2} \qquad (1)$$

Equations for $\Delta R_{iso}(n)$, $\Delta R_{iso}(n)$ and $\Delta R_{iso}(n)$ are given in (Hellenkamp et al. 2018). Due to the position of our labels on the DNA, we expect that the medium between them will be composed almost exclusively of buffer. We thus assume a refractive index of n=1.35±0.02, which leads to $\Delta R_{iso}(n)$=0.01*R_{iso}. We further assumed an error of 4% for the donor quantum yield based on the uncertainties in the donor lifetime and radiative rate constant, leading to $\Delta R_{iso}(\phi)$=0.01*R_{iso}. The value of $\Delta R_{iso}(J)$=0.025%*R_{iso} was adopted from (Hellenkamp et al. 2018). These uncertainties lead to ΔR_{iso}=0.03*R_{iso}=2Å for the construct DNA_{Tamra}.

<u>Data for simulations: lifetime and time-resolved anisotropy</u>

Data for the donor in absence of the acceptor was obtained from the donor-only population of the smFRET measurements. All donor photons after donor excitation (531nm) of the donor-only population (uncorrected stoichiometry >0.9 and more than 100 photons) were combined in one TCSPC histogram. Data for the acceptor can be gained either from the acceptor-only population (uncorrected stoichiometry <0.25 and more than 70 photons) or from the FRET population (as defined above), because the acceptor photons after acceptor excitation (640nm) are not affected by the FRET process. Results for both species were generally in good agreement, so a mean value was taken.

Fluorescent lifetime, rotational correlation time and residual anisotropy were determined by iterative re-convolution fitting of the parallel and perpendicular decays (Supplementary Figure 22). Concentrated solutions of crystal violet or malachite green (two fluorescent dyes with lifetimes in the picosecond range) were used to record the instrument response function (IRF) of the green and red channels, respectively.

In a first step, the fluorescence lifetime was determined from the combined TCSPC histogram *D(t)*, which adds the data from parallel ($D_{\parallel}$) and perpendicular ($D_{\perp}$) detection channels according to

$$D(t) = (1 - 3l_2) \cdot G \cdot D_{\parallel}(t) + (2 - 3l_1) \cdot D_{\perp}(t) \tag{2}$$

with *G*, l_1 and l_2 being the correction factors described above. For Atto550 a mono-exponential and a bi-exponential model function were needed to describe the acceptor Alexa647. Iterative re-convolution of the model decay with the recorded IRF was used to fit the TCSPC histogram *D(t)*, additionally considering a constant background contribution. For the simulations, the amplitude weighted average lifetime of the two donor components was used. The value for the donor lifetime given in Supp. Table S5 represents the average of the fit results from DNA, DNA+F2 and DNA+F1F2, because they were in agreement.

In the second step, the anisotropy information was analyzed in a global fit of the TCSPC data from the parallel ($D_{\parallel}$) and perpendicular ($D_{\perp}$) detection channels by iterative re-convolution. The model functions were

$$M_{\parallel}(t) = \frac{1}{G} \cdot I(t) \cdot [1 + (2 - 3l_1) \cdot r(t)] \tag{3a}$$

$$M_{\perp}(t) = I(t) \cdot [1 - (1 - 3l_2) \cdot r(t)] \tag{3b}$$

with *I(t)* being the biexponential fluorescence intensity decay and *r(t)* being the model function for the anisotropy decay, given by one of the following functions, depending on the complexity of the data.

$$r(t) = (r_0 - r_\infty) \cdot e^{-\frac{t}{\rho}} + r_\infty \tag{4a}$$

$$r_{tumb}(t) = \left[(r_0 - r_\infty) \cdot e^{-\frac{t}{\rho}} + r_\infty \right] \cdot e^{-\frac{t}{\rho_{tumb}}} \tag{4b}$$

With the fundamental anisotropy r_0, the residual anisotropy r_∞, the rotational correlation time of the dye ρ and the tumbling time of the DNA ρ_{tumb}. In order to reduce the number of free parameters, the lifetimes and their fractions were fixed to the results from the lifetime fit. A constant background contribution was also assumed. The simple anisotropy model was used for the donor data of all datasets. For all acceptor data, the anisotropy model with two lifetimes and two rotational correlation times was used.

As in the case of the donor lifetime, the values given in Supp. Table S5 for the rotational correlation times of donor or acceptor represent the average of the fitting results from DNA, DNA+F2 and DNA+F1F2, because their difference was close to the expected uncertainty. The value for the residual anisotropy of the donor was taken from the fit of DNA+F1F2 (Supplementary Figure 22), because for this dataset, the DNA tumbling time was sufficiently long to play no role. Thus, the residual anisotropy should be most

accurate if determined from this dataset, where a simple model is sufficient. The value for the residual anisotropy of the acceptor was taken as the average of the fit results from DNA, DNA+F2 and DNA+F1F2. In the presence of protein, the situation becomes more complicated than our simulation can describe, with two acceptor states due to PIFE [5]. Even so, on a long timescale, the anisotropy is expected to decay to the lower value of the unrestricted state, which is likely to be dominant in the absence of protein.

Static and dynamic FRET lines

In order to test for conformational dynamics, static and dynamic FRET lines were simulated [6] and overlayed with the plot of FRET efficiency vs. burst-wise donor lifetime (Supp. Fig. S8a). The required donor lifetime was obtained from the donor-only population of the measurement, the Förster radius for DNA_{AT550} was 70 Å and the apparent linker length of 7 Å was determined from the measurement of ligated DNA by fitting the fluorescence decay by a Gaussian distance distribution. For the dynamic FRET line, interconversion was assumed between the state of free DNA and the most kinked state observed in the measurement of DNA+F1F2.

Burst variance analysis

Burst variance analysis (BVA) tests for dynamics by comparing the shot-noise-limited standard deviation of the mean proximity ratio within a burst to the observed standard deviation [7]. Here, the E_{PR} fluctuations are assessed via splitting the burst into chunks of 10 photons and then calculating the E_{PR} for each chunk and the standard deviation over all chunks (burst-wise σ of E_{PR}). A binned standard deviation is calculated over all chunks in all bursts in an E_{PR}-bin (bin size of E_{PR} = 0.05, >50 bursts per bin). The σ of E_{PR} expected from shot-noise for each chunk is calculated and then compared to the binned σ of E_{PR}. The bursts with σ of E_{PR} values close to the expected σ (those that lie on the black line) indicate static FRET events, whereas those which are above the black line indicate dynamic FRET events (Supp. Fig. S8b).

Time window analysis

Dynamic interconversion can be overseen if it takes place on the faster timescale than the observation time window [8]. To observe this dynamics, the time binning of the data was varied from 0.25 to 10 ms. If dynamics is present, the shape of E histograms changes upon increase of the time window length. Therefore, to provide additional evidence for conformational dynamics, FRET efficiency was plotted at 0.25, 0.5, 1, 2, 5, and 10 ms observation time windows (Supp. Fig. S8c). A threshold of 50 photons was set to exclude the time windows with lower number of photons.

FRET-FCS of the acceptor only species

FRET-FCS [9,10] analysis was employed to observe the diffusion time τ_D for DNA_{Tamra}, DNA_{Tamra}+F1, DNA_{Tamra}+F2 (Supplementary Figure 4), DNA+PARP-1, DNA+PARP-1+niraparib (Supp. Fig. S9), and for all XRCC-1 data (Fig. 7c,d, Supplementary Figure 15, 16). Additionally, to demonstrate the reasoning behind the choice of protein concentration in smFRET measurements (methods), FRET-FCS was employed to observe the diffusion time τ_D in titration of F2 and F1F2 to nicked DNA (Supplementary Figure 20). For this, the red parallel and red perpendicular channels (AA_{par}, AA_{per}) of the respective *FRET populations* were correlated: entire populations for free DNA_{Tamra}, DNA_{Tamra}+F1, DNA_{Tamra}+F2 (Supplementary Figure 4), DNA, DNA+XRCC1, DNA+F2+XRCC1 (Fig. 7d), DNA+F1F2+XRCC1 and DNA+PARP-1+XRCC1 (Supplementary Figure 15) and subpopulations, 0<E<0.59 for DNA+PARP-1 and 0<E<0.55 for DNA+PARP-1+niraparib (Supplementary Figure 10), 0<E<0.34/0.63<E<0.69/0.86<E<1 for DNA+F2+XRCC1 (Supplementary Figure 17), 0<E<0.66 for DNA+F1F2 in 200mM salt buffer (Supp. Fig.20) with 0.3<S<0.8 for all datasets.

The obtained autocorrelation functions were fit with a one component FCS diffusion model with an additional term, accounting for acceptor photophysics τ_{phot} with fraction A_{phot}:

$$G(\tau) = \frac{1}{N\sqrt{8}}\left[1+\frac{\tau}{\tau_D}\right]^{-1}\left[1+\frac{\tau}{p^2\tau_D}\right]^{-1/2}\left[1+\frac{A_{phot}}{1-A_{phot}}\,exp\left(-\frac{\tau}{\tau_{phot}}\right)\right] \quad (5)$$

where, N is the average number of the molecules in the effective observation volume, τ_D is the diffusion time (or dwell time in the observation volume, depends on radius ω_0 of the observation volume, $\tau_D = \omega_0^2/4D$, where D is the diffusion coefficient, Ries and Schwille 2012), p is the ratio of the lateral and axial dimensions of the observation volume. Standard error of the mean (SEM) was calculated by splitting the respective dataset into 4 to 6 equal time periods. Every time period was again subjected to FRET-FCS analysis and the resulting diffusion times were then used for SEM calculation.

Additionally, FRET-FCS analysis was employed to observe the effect of acceptor photophysics upon PARP-1 binding to DNA (Supplementary Figure 9). To this end, the red parallel and red perpendicular channels of the *acceptor only species* (AA_{par}, AA_{per}), selected using a stoichiometry cut 0<S<0.23, from smFRET measurement) for ligated DNA, DNA, DNA+F2, DNA+F1F2 and DNA+PARP-1 were correlated. The obtained autocorrelation functions were fit with the same model described above model. The returned , τ_D and τ_{phot}were fixed when fitting the fFCS curves (methods).

Supplementary figures

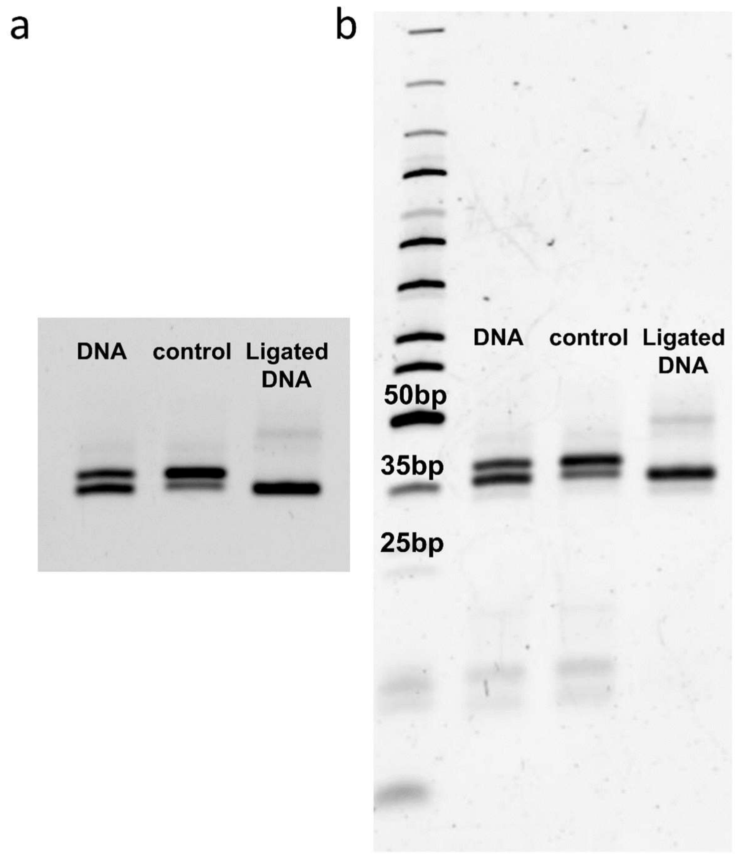

Supplementary Figure 1: Ligation efficiency of the nick was tested using gel electrophoresis.
Gel electrophoresis of nicked and ligated DNA. DNA: phosphorylated nicked DNA before ligation. Ligated DNA: the same DNA after ligation with T4 DNA ligase. Control: the same DNA, treated as for the ligated sample except that water was added instead of ligase. Panel a shows a FRET image, i.e. only double-labeled bands are visible. Panel b shows the Sybr Gold™ staining. The two bands in the cases of the DNA and control samples are presumably due to the phosphorylated and unphosphorylated DNA fractions, the difference in the relative intensity between DNA and control could be explained by an additional phosphorylation step in the control sample (Methods). The gel demonstrates that ligation was very efficient. This experiment was repeated 3 times.

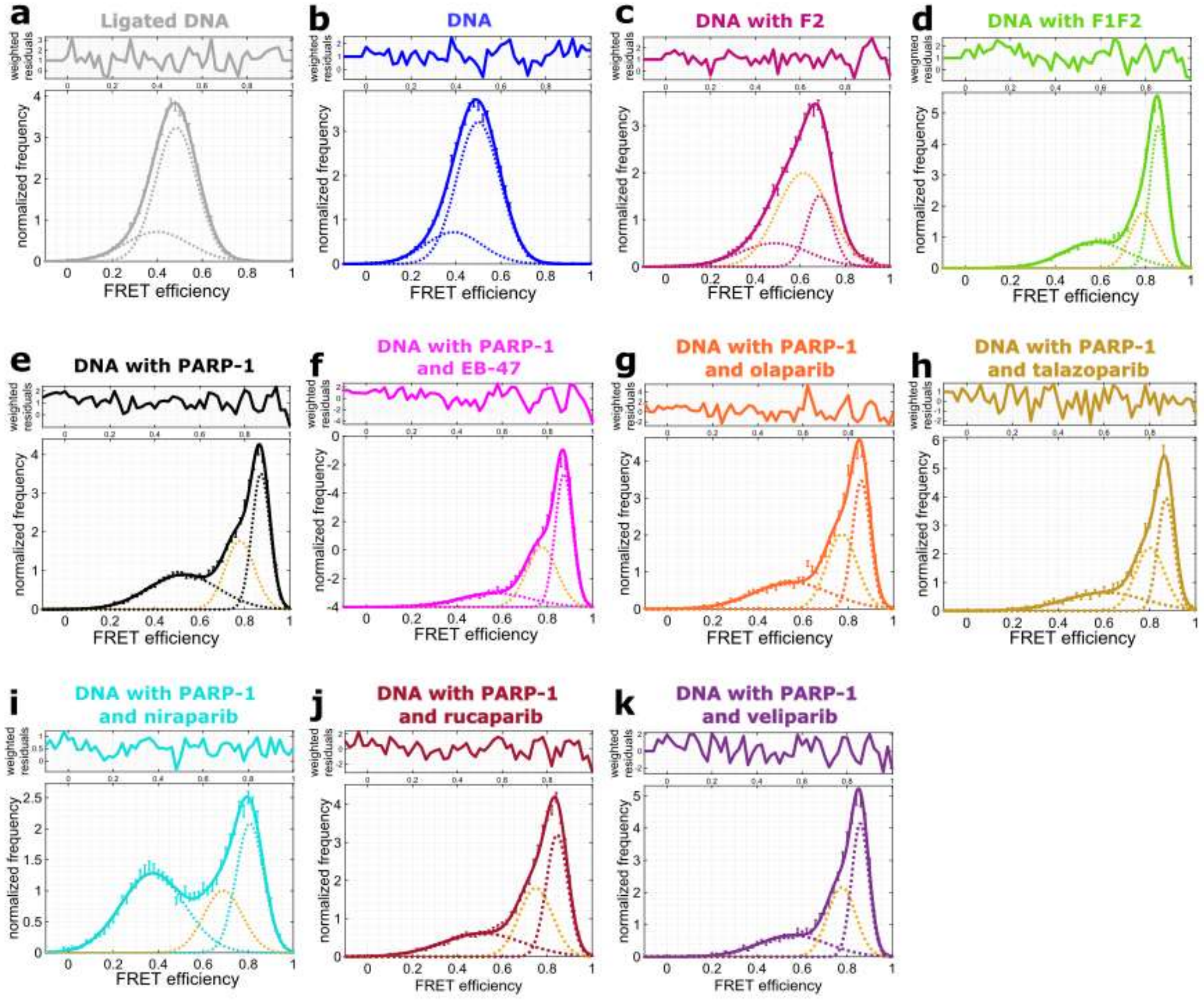

Supplementary Figure 2: Quantification of FRET efficiencies.

Gaussian fits of the smFRET efficiency histograms shown in Figs. 1, 2 and 6. **a:** ligated DNA alone; **b:** DNA alone: **c:** DNA in presence of 10µM F2; **d:** DNA in presence of 1µM F1F2; **e:** DNA in presence of 1µM full-length PARP-1; **f:** DNA in presence of 1µM PARP-1 and 200µM of either EB-47 (**f**), olaparib (**g**), talazoparib (**h**), niraparib (**i**), rucaparib (**j**) or veliparib (**k**). The data was fitted with a two Gaussians (a and b) or with the sum of three Gaussians (b-k), where in each case the third Gaussian (yellow) accounts for a broad distribution of different structural species. All parameters were freely adjustable during fitting. Fit results are summarized in Supp. Table. S1. The error bars show the standard error of the mean for the FRET efficiency of the single molecules contributing to the same bin.

a

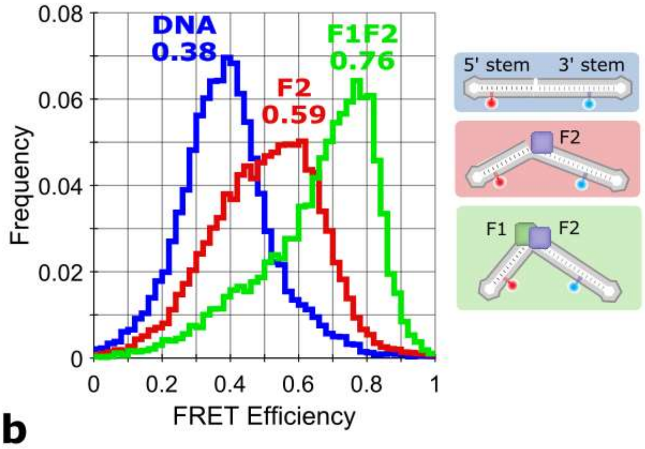

b

	A_1	E_1	σ_1	A_2	E_2	σ_2	R^2
**DNA**	1.00	0.38	0.11				0.986
**lig. DNA**	1.00	0.38	0.12				0.988
**F2**	0.40	0.38 (fix)	0.11	0.60	0.59	0.10	0.995
**F1F2**	0.51	0.57	0.17	0.49	0.76	0.08	0.985

Supplementary Figure 3: DNA conformation during nick recognition probed by smFRET with an alternative DNA construct with TAMRA as donor (DNA_{Tamra}).

In order to check the influence of dye molecules on the obtained results, experiments were repeated using an alternative DNA construct with TAMRA as a donor [3]. **a:** smFRET efficiency histogram of nicked DNA (blue) and nicked DNA in presence of either 10µM F2 (red) or 1µM F1F2 (green). The indicated peak FRET efficiencies were obtained by Gaussian fitting. **b:** Results of Gaussian fitting of smFRET efficiency histograms where *A*: area fraction, *E*: mean FRET efficiency, *σ*: standard deviation of the Gaussian. FRET efficiencies of the species shown in A are highlighted. While the specific FRET efficiencies are slightly different the general effect observed is comparable to that of the original DNA construct.

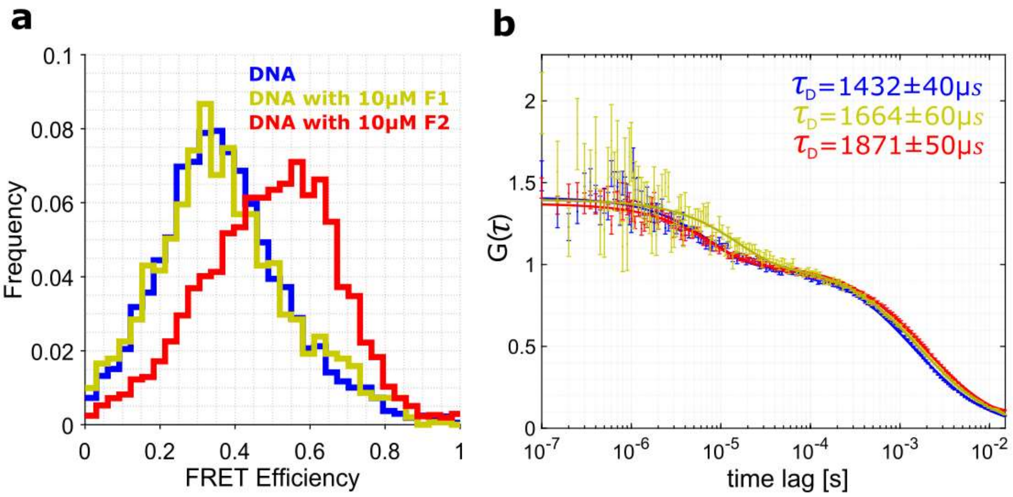

Supplementary Figure 4: Comparison of single zinc finger F1 and F2 binding.

In order to compare the effect of the binding of single zinc finger F2 and single zinc finger F1, either one was added at 10µM concentration to the alternative nicked DNA sample using the Tamra dye as a donor (Methods). **a:** smFRET efficiency histogram of nicked DNA (blue) and nicked DNA in presence of either 10µM F2 (red) or 10µM F1 (yellow). The FRET histogram in presence of F1 overlaps with that of free DNA, whereas a shift towards higher FRET efficiency is observed in presence of F2. **b:** FRET-FCS results for data in a, respectively, using the same color coding; the extracted diffusion times are displayed in the upper right corner of the FRET-FCS plot and the complete fit results are shown in Supp. Table S6. The increased diffusion time for DNA in presence of either F1 or F2, show that the majority of DNA molecules are bound to protein for both cases. The shorter diffusion time in presence of F1 then that in presence of F2 indicates that more free DNA is present in the former case and thus the average diffusion time is decreased, in agreement with the reported lower affinity of F1 to nicked DNA [12,13]. The error bars show the standard error of the mean the computed molecules contributing to the same bin.

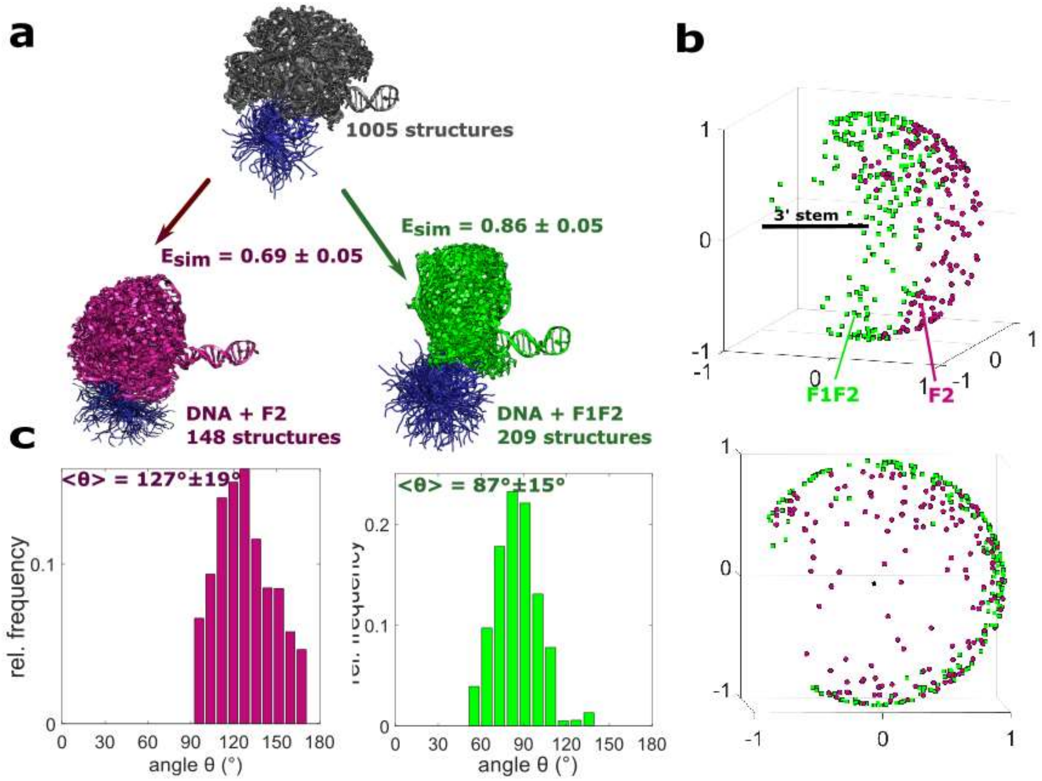

Supplementary Figure 5: Selection and analysis of structures in accordance with experiment.

a: Structures in agreement with the experimental FRET efficiencies (see Fig. 2) were selected from the ensemble of DNA+F2 (1005 structures, 100 of which are shown here in grey) as follows: *E*=0.69 for DNA+F2 (148 selected structures shown in magenta), *E*=0.86 for DNA+F1F2 (209 selected structures shown in green). An uncertainty of ΔE=±0.05 was assumed in all cases. **b:** Combined representation of the selected structures from DNA+F2 ensemble (1005 structures) shown in A. All structures are aligned with respect to the 3' stem, the axis of which is represented here by a black line. The colored shapes indicate the direction of the 5' axis for each selected structure: magenta circles for DNA+F2; green squares for DNA+F1F2. Two different views of the 3D plot are shown, a side view (top) and a view along the 3' axis (bottom). **c:** Kinking angle distributions for the selected structures in a: DNA+F2 (magenta, left hand histogram), DNA+F1F2 (green, right hand histogram). The mean and standard deviation of the angles are indicated for each histogram.

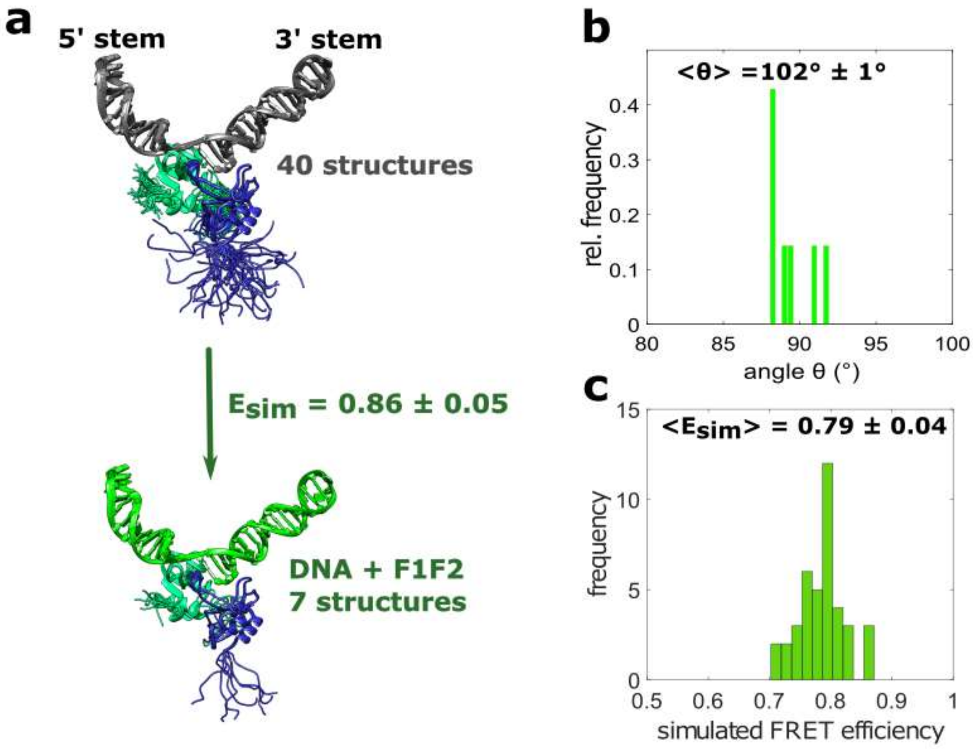

Supplementary Figure 6: Simulated structure of nicked DNA bound by F1F2.

a: Ensemble of 40 model structures for the DNA ligand bound to F1F2. Both stems of the DNA are modeled in B-form. Contacts between F1 and the 5' stem, between F2 and the 3' stem and between the two zinc fingers were modeled, and as well orientational restraints from residual dipolar coupling data were included in the calculations of these models, using values measured in the context of a similar (1nt gapped) DNA ligand by NMR [1]. The kinking angle of the DNA is defined as the angle between the axes of the two stems. DNA in grey, F1 in light green, F2 in purple, zinc ions in brown. **b:** Histogram of kinking angles for the structures of the DNA bound to F1F2 shown in A. The mean and standard deviation of the angle distribution is shown. **c:** Histogram of simulated FRET efficiencies for the 40 structures of the DNA bound to F1F2 shown in A. Mean and standard deviation are given above the plot.

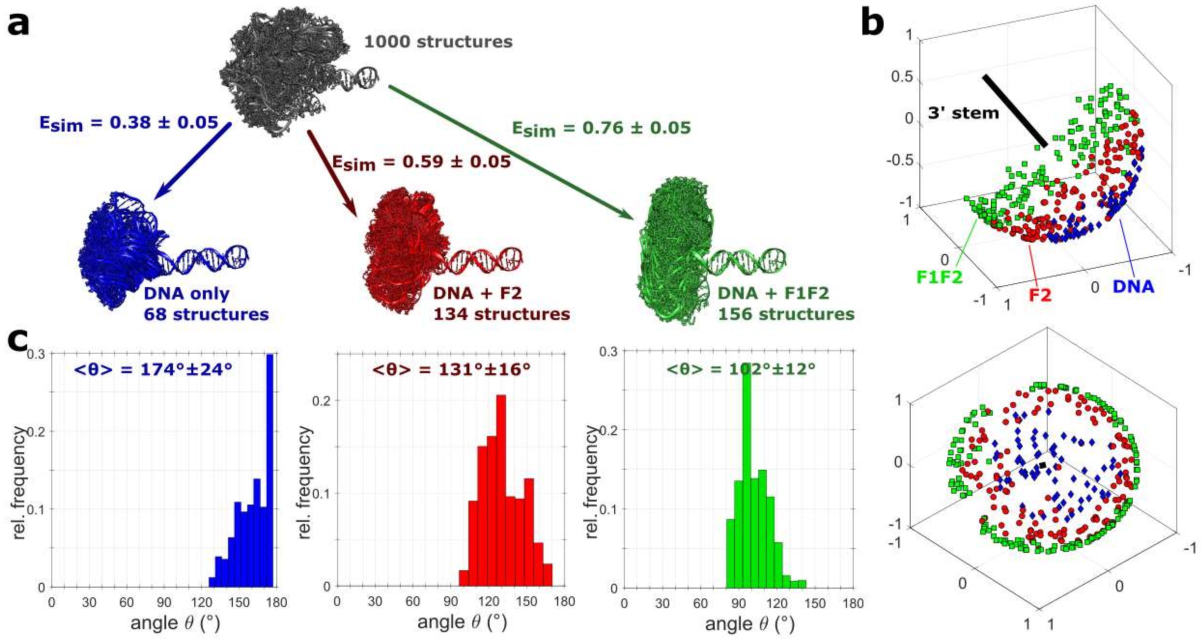

Supplementary Figure 7: Selection and analysis of structures in accordance with experiment for the alternative DNA construct with TAMRA as donor (DNA_{Tamra}).

a: Structures in agreement with the experimental FRET efficiencies (see Fig. 2) were selected from the ensemble of DNA (1000 structures, 100 shown in grey): *E*=0.38 for free DNA (68 structures in blue), *E*=0.59 for DNA+F2 (134 structures in red), *E*=0.76 for DNA+F1F2 (156 structures in green). An uncertainty of ΔE=±0.05 was considered in all cases. **b:** Combined representation of the selected structures shown in a. All structures are aligned with respect to the 3' stem. The axis of the 3' stem is represented by a black line. The colored shapes indicate the direction of the 5' axis for each selected structure: blue diamonds for free DNA; red circles for DNA+F2; green squares for DNA+F1F2. Two different views of the 3D plot are shown, a side view (top) and a view along the 3' axis (bottom). **c:** Kinking angle distributions for the selected structures in a. From left to right: free DNA (blue), DNA+F2 (red), DNA+F1F2 (green). The mean and standard deviation of the angles are indicated for each histogram [3].

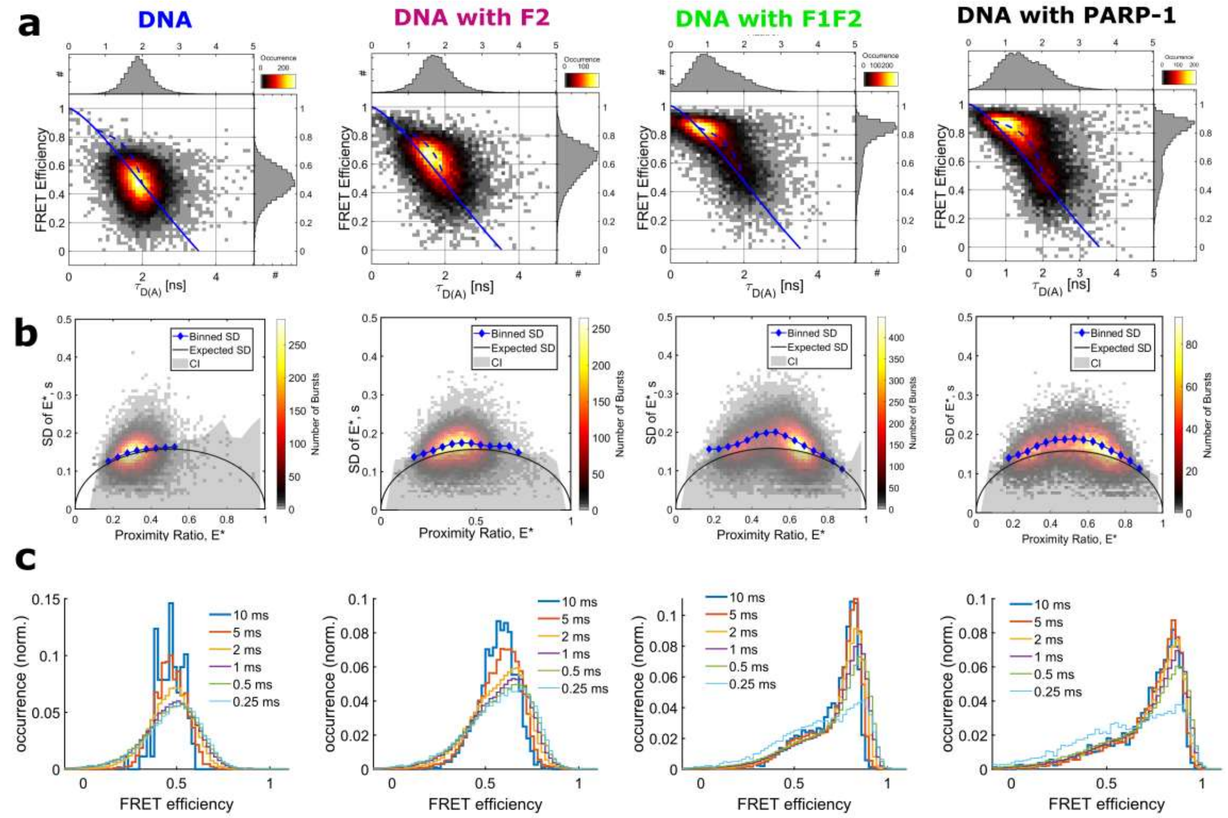

Supplementary Figure 8: **Qualitative analysis of dynamics for DNA, DNA+F2, DNA+F1F2 and DNA+PARP-1 datasets.**

a: E vs. TauD plots with static (solid) and dynamic (dashed) lines indicate dynamics for DNA with F2, DNA with F1F2 and DNA with PARP-1 where the respective population deviates from the static FRET line. **b:** Burst variance analysis (BVA) results show the dynamics in DNA with F2, DNA with F1F2 and DNA with PARP-1 where the binned standard deviation (blue diamonds) is above the expected standard deviation (black solid line). **c:** Time window analysis results show the dynamics in DNA with F2, DNA+F1F2 and DNA with PARP-1 where the FRET efficiency histograms change their shape upon increase of the time window. All three analysis schemes (supplementary methods) indicate the presence of the dynamic interconversion in the cases of DNA bound by PARP-1 or its fragments, whereas in contrast for the DNA alone no dynamics is observed.

a

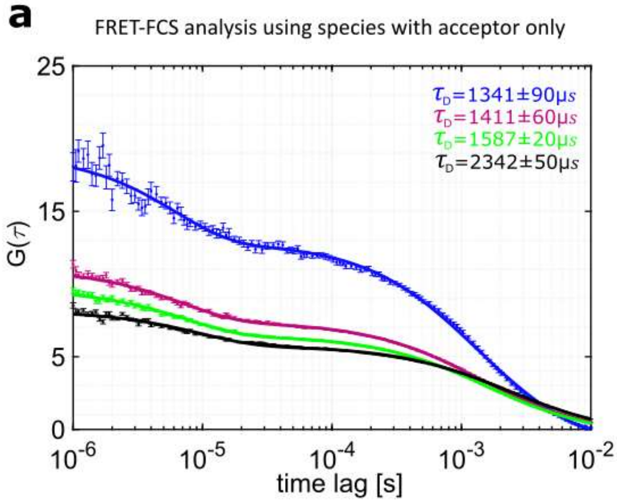

b

Data Set	N	τ_D [μs]	τ_{phot} [μs]	A_{phot}	y_0	χ^2
DNA	0.024	1341	6.08	0.43	-1.52	1.60
DNA with F2	0.052	1411	7.01	0.47	-0.30	1.07
DNA with F1F2	0.046	1587	7.38	0.47	-0.37	0.89
DNA with PARP-1	0.058	2342	9.58	0.40	-0.29	1.86

Supplementary Figure 9: Investigation of the acceptor blinking time τ_{phot}.

a: The respective fitted autocorrelation functions of acceptor only species in the red channel: blue – DNA, magenta – DNA+F2, green – DNA+F1F2 and black – PARP-1 which was correlated additionally for better comparison. **b:** Corresponding fit results for autocorrelation functions of the acceptor only species in the red channel shown in b. The diffusion time τ_D and the acceptor blinking component τ_{phot} are used in the fFCS (Methods) as fixed parameters. An increased τ_{phot} is observed for DNA bound by protein datasets . The increase of τ_{phot} here occurs most probably due to protein induced fluorescence enhancement (PIFE) effect [5] as our acceptor dye is attached to 5' stem, exactly where the PARP-1 or its fragments bind. The error bars show the standard error of the mean the computed molecules contributing to the same bin.

a

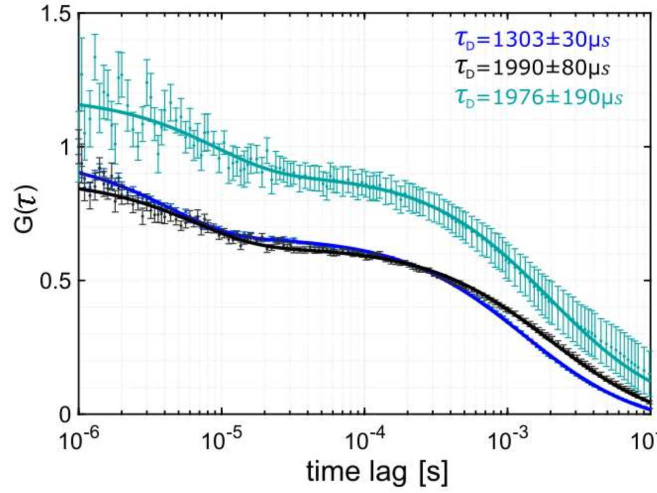

b

	N	τ_D [μs]	τ_{phot} [μs]	A_{phot}	y_0	χ^2
**DNA**	0.027	1303	4.30	0.30	-0.96	2.50
**DNA with PARP-1**	0.039	1990	6.48	0.27	-0.72	0.78
**DNA with PARP-1 and Nirapairb**	0.028	1976	8.88	0.24	-013	0.34

Supplementary Figure 10: Investigation of diffusion time τ_D for low FRET species of DNA+PARP-1 and DNA+PARP-1+Niraparib.

a: Fitted autocorrelation functions of FRET sub-species: blue – DNA, grey – low FRET population of the DNA+PARP-1 (0 – 0.59), turquoise – low FRET population of the DNA+PARP-1+niraparib (0 – 0.55). For the fits data from the acceptor fluorescence after acceptor excitation was used. **b:** Corresponding fit results for the respective autocorrelation functions: N is the average number of molecules in observation volume, τ_D is the diffusion time, τ_{phot} is a time scale characterizing acceptor blinking (compare to Supplementary Figure 7), A_{phot} is the fraction of the blinking and y_0 is the offset (Methods). An increased τ_{phot} is observed for DNA bound by protein datasets. The error bars show the standard error of the mean the computed molecules contributing to the same bin.

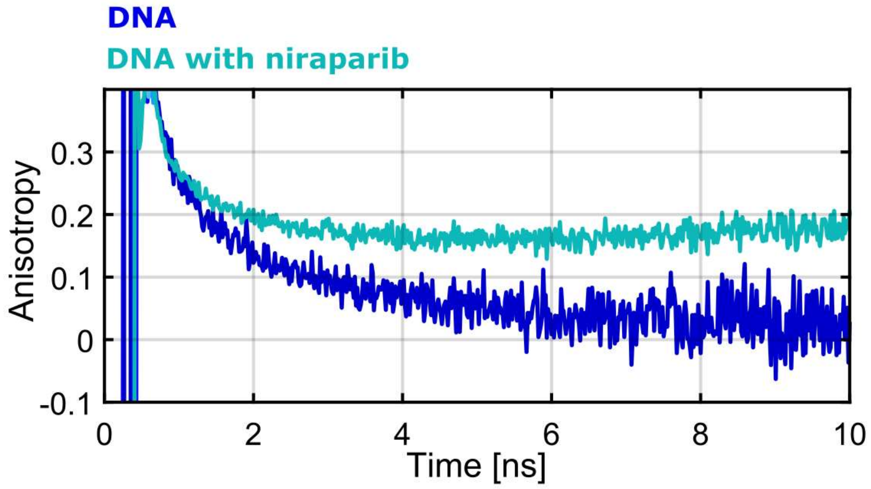

Supplementary Figure 11: Donor anisotropy decays for DNA (blue) and DNA with Niraparib (turquise)
The anisotropy decay (Supplementary methods) for DNA in presence of niraparib indicates a strong hindrance of the donor dye attached DNA (residual anisotropy r_∞ of DNA with niraparib is higher than that of DNA in absence of niraparib), whereas the donor dye without niraparib rotates freely (r_∞ approaches 0). This hindrance of the donor dye results in slightly lower FRET efficiencies for DNA with PARP-1 and niraparib data (Fig. 6 B, turquoise, Supplementary Figure 2, Supp. Table S1).

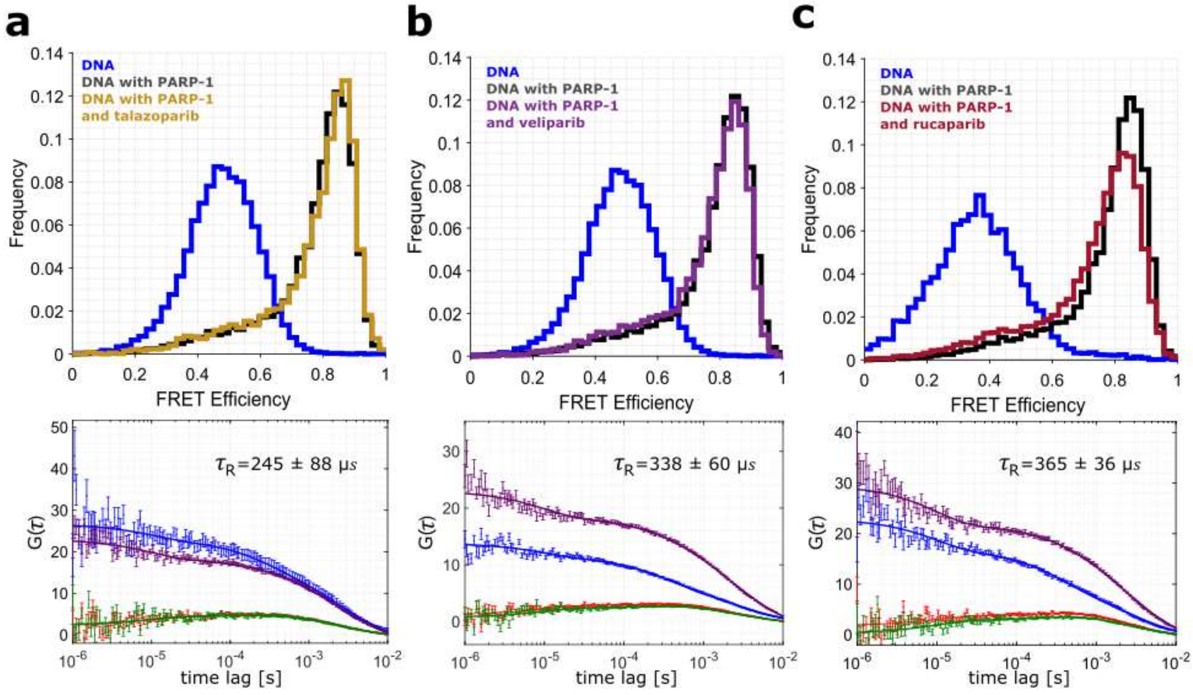

Supplementary Figure 12: DNA conformation probed by smFRET in presence of PARP-1 and additional PARP inhibitors.

a: smFRET efficiency histogram of nicked DNA (blue) and nicked DNA in presence of either PARP-1 (grey, same histogram as in Fig.1) or PARP-1 and talazoparib (gold), a class I PARP-1 inhibitor **b**: (upper panel) smFRET efficiency histogram of nicked DNA (blue) and nicked DNA in presence of either PARP-1 (grey) as well as in presence of PARP-1 and veliparib (violet), a class III PARP-1 inhibitor. **c:** (upper panel) smFRET efficiency histogram of nicked DNA (blue), nicked DNA in presence of PARP-1 (grey), as well as nicked DNA in presence of PARP-1 and veliparib (burgundy), a class III PARP-1 inhibitor. Note, since rucaparib has similar effect as niraparib (Supplementary Figure 13), unlike rest of the PARPi, for a better comparison the DNA only data in (c) is presented in presence of rucaparib. Lower panels show dynamic analysis of the respective samples using fFCS, namely DNA in presence of PARP-1 and inhibitor: talazoparib (a), veliparib (b) and rucaparib (d), where violet and blue are the auto- correlation and, green and red are the cross-correlation functions, respectively. In the top right corner of the fFCS plot the resulting relaxation times are displayed. The error bars in fFCS fits show the standard error of the mean for the computed molecules contributing to the same bin.

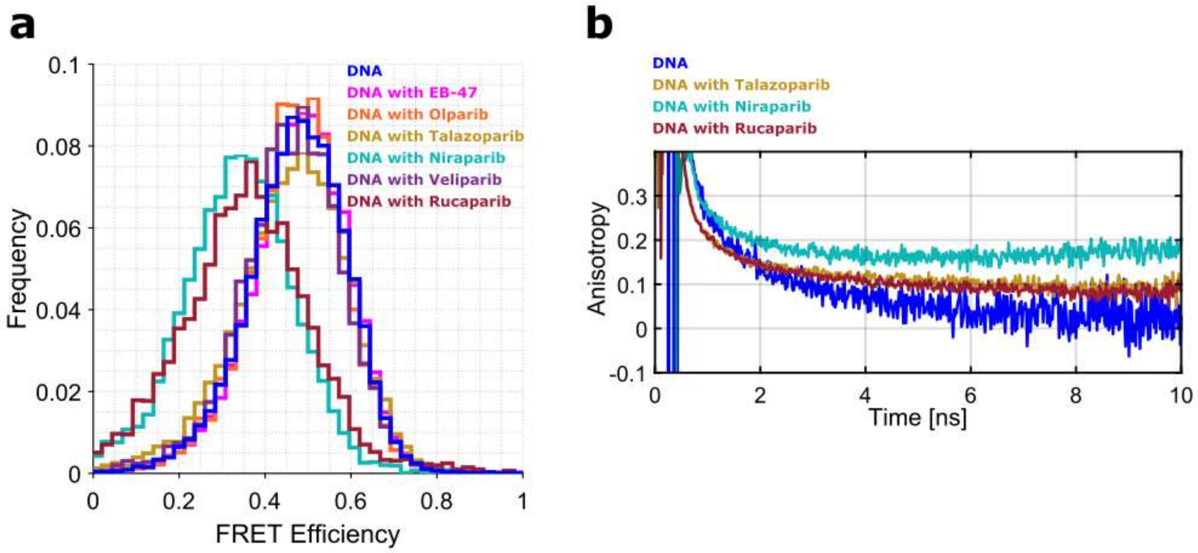

Supplementary Figure 13: smFRET measurements of DNA in presence of different PARP inhibitors. Niraparib and rucaparib are the only PARPi which affect the DNA FRET histogram. In all figures the FRET efficiency histogram of DNA alone was used apart from niraparib and rucaparib where the histogram of DNA in presence of these inhibitors was used. **a:** smFRET efficiency histogram of nicked DNA and nicked DNA in presence of all PARPi used in this study. **b:** The anisotropy decay (Supplementary methods) for nicked DNA alone, nicked DNA in presence of niraparib (same as in Supplementary Figure 9), talazoparib and rucaparib. While niraparib hinders the rotation of the donor dye (residual anisotropy r_∞ of DNA with niraparib is substantially higher than that of DNA in absence of niraparib or in presence of rucaparib), rucaparib does not seem to affect the rotational motion of the donor dye and similiar time-resolved fluorescence anisotropy as those PARPi that do not affect the FRET efficiency histogram in a (here: data for talazoparib are shown for comparison). The data for rucaparib can be interpreted as an effect on the extinction coefficient of the donor dye. This change in extinction coeffcient of the donor dye results in slightly lower FRET efficiencies for DNA with rucaparib data (a).

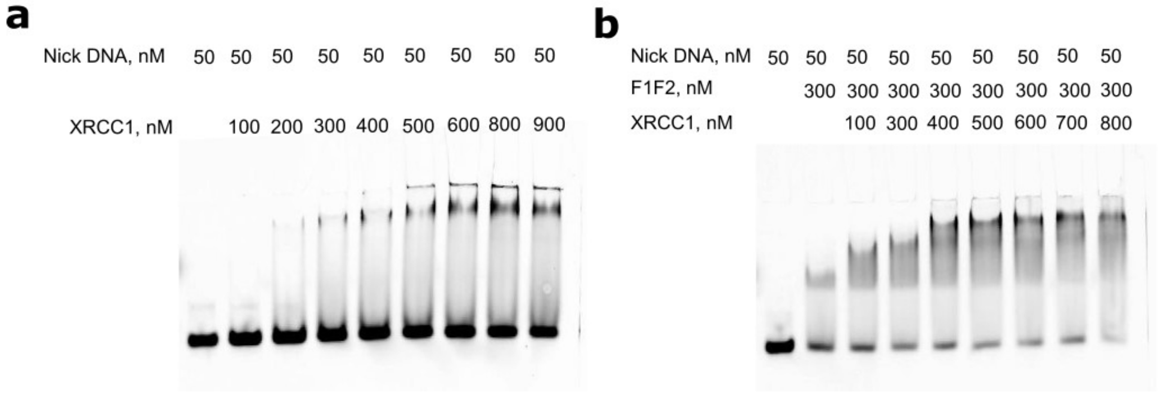

Supplementary Figure 14: XRCC1 binding to DNA ligand in presence and absence of PARP-1 fragment F1F2.

Binding of recombinantly expressed XRCC1 protein (Methods) to the DNA ligand was studied using EMSA a) Binding of XRCC1 to DNA ligand in absence of PARP-1. 50nM nicked DNA with an increasing amount of XRCC-1 (100-800nM) shows XRCC-1 binding. **b:** Binding of XRCC1 in presence of F1F2**.** EMSA for 50nM nicked DNA in presence of 300nM F1F2 with an increasing amount of XRCC-1 (100-900nM). Comparison of a) and b) shows that XRCC1 binding is enhanced by the presence of F1F2. Both gels were imaged using the acceptor fluorescence (Alexa647) of DNA_{Tamra} . This experiment was repeated 3 times.

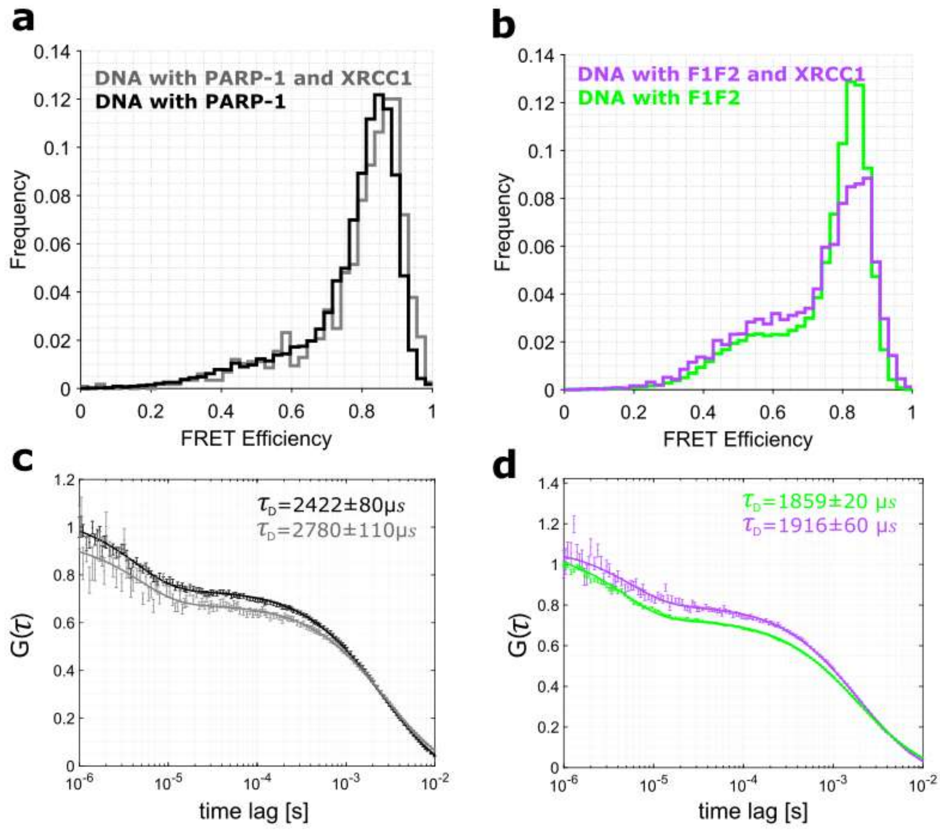

Supplementary Figure 15: smFRET measurements in presence of both PARP-1 and XRCC1.

a: smFRET efficiency histograms of nicked DNA in presence of 1µM PARP-1 (black), and nicked DNA in presence of 1µM PARP-1 and 1µM XRCC1 (grey), indicate that XRCC1 does not influence DNA kinking. **b**: smFRET efficiency histograms of nicked DNA in presence of 1µM F1F2 (green), and nicked DNA in presence of 1µM F1F2 and 1mM XRCC1 (violet), are in good agreement indicating that XRCC1 also does not influence DNA kinking in presence of F1F2. **c** and **d**:FRET-FCS results for data in a and b, respectively, using the same color coding; the extracted diffusion times are displayed in the upper right corner of the FRET-FCS plot and complete fit results are shown in Supp. Table S6. For PARP-1 a clear increase in diffusion time is observed in presence of XRCC1 (c). In summary, these experiments show, that the observed complexes are bound to XRCC1 and that XRCC1 binding does not alter the observed kinked state. The error bars show the standard error of the mean the computed molecules contributing to the same bin.

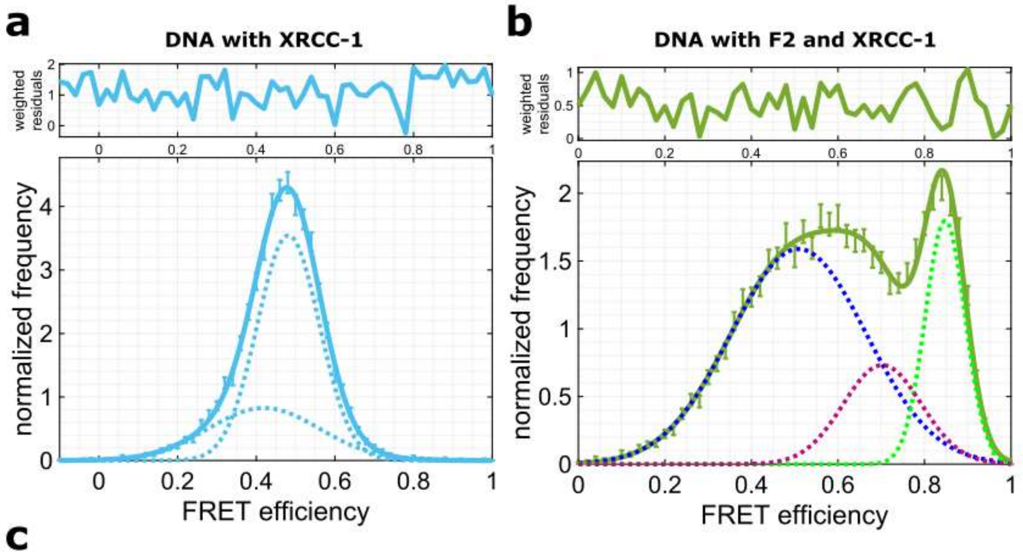

c

	A_1	E_1	σ_1	A_2	E_2	σ_2	A_3	E_3	σ_2	R^2
**DNA with XRCC-1**	0.70	0.48	0.08	0.29	0.42	0.14	-	-	-	1.10
**DNA with F2 and XRCC-1**	0.62	0.51	0.21	0.17	0.70	0.09	0.21	0.85	0.46	1.13

Supplementary Figure 16: Quantification of FRET effcicency for XRCC-1 data.

Gaussian fits of the smFRET efficiency histograms shown in Fig. 7a,c for DNA+XRCC1 (**a**) and DNA+F2+XRCC1 (**b**). The corresponding fit results are shown below Gaussian fits (**c**), where *A*: area fraction, *E*: mean FRET efficiency, σ: standard deviation of the Gaussian. DNA conformation is not affected by XRCC1 binding as it results in FRET efficiency of 0.48, identical or very similar to that of ligated (0.48) or nicked DNA (0.50, Supp. Table S1). DNA-F2-XRCC1 fit results in three FRET efficiency values which are almost identical to the values of linear DNA, DNA+F2, DNA+F1F2 (0.50, 0.69, 0.86 respectively in Supp. Table S1). The error bars show the standard error of the mean for FRET efficiency of the single molecules contributing to the same bin.

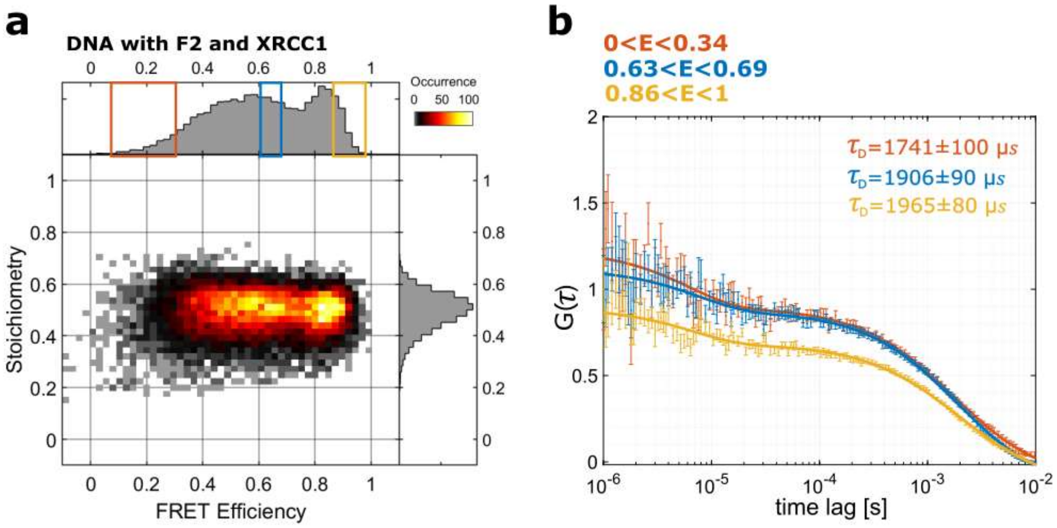

Supplementary Figure 17: Further analysis of smFRET measurements of DNA ligand in presence of F2 and XRCC1.

a: 2D plot of stoichiometry versus smFRET efficiency for DNA+F2+XRCC1, together with the respective 1D projections. In the 1D smFRET efficiency projects FRET efficiency regions are marked in order to perform a species selective burst selection for FRET-FCS analysis shown in b. **b:** Fitted FRET-FCS autocorrelation functions of specific smFRET ranges: low FRET region 0<E<0.34 marked with orange, middle FRET region 0.63<E<0.0.69 marked with blue and high FRET region 0.86<E<1 marked with yellow. The computed diffusion times are displayed in the upper right corner and complete fit results are shown in Supp. Table S6.. When compared to the computed diffusion time for free DNA ($\tau_D = 1303$±26μs, Supplementary Figure 8) the computed diffusion time for all three smFRET species are considerably higher, showing that all three states of the DNA ligand (linear, pre-kinked, kinked) are states with protein bound. One should also note, that for all states the computed diffusion times are longer than that observed for F2 bound to the DNA ligand, showing XRCC1 binding for all states. The error bars show the standard error of the mean the computed molecules contributing to the same bin.

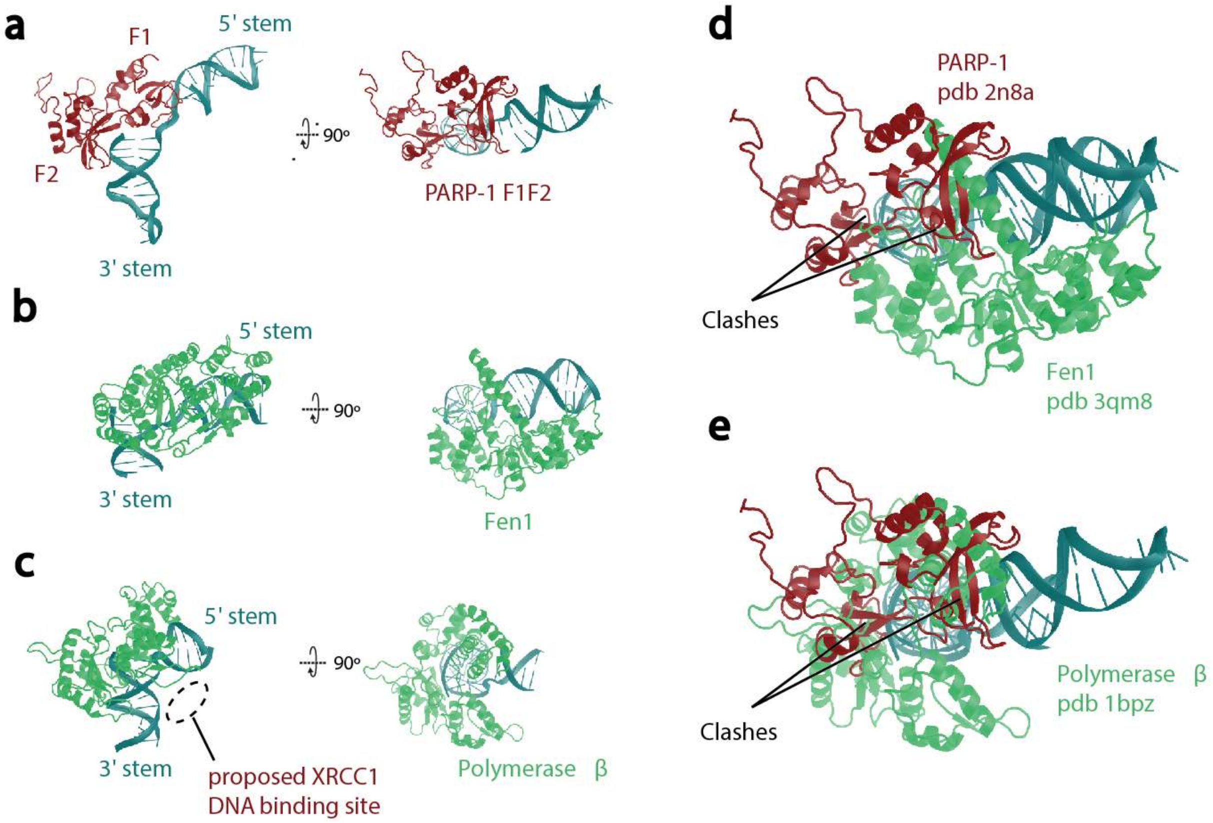

Supplementary Figure 18: Structural comparison suggests a mutually exclusive binding of PARP-1, Fen1 or DNA Polymerase β at DNA damage sites.

Structures of **(a)** human PARP-1 F1F2 bound to a DNA containing a single-stranded gap (pdb 2N8A [10.1016/j.molcel.2015.10.032]) **(b)** human Fen1 bound to flap DNA substrate (pdb 3Q8M [10.1016/j.cell.2011.03.004], Tsutakawa et al. 2011)) and **(c)** human DNA polymerase β bound to a DNA ligand containing a single-stranded nick (pdb 1BPZ [10.1021/bi9703812d], Sawaya et al. 1997) were superimposed by using their respective 3' DNA stem, and are shown here in two different orientations. Panels **d** and **e** show a superimposition of the PARP-1 structure with the Fen1 and DNA polymerase β structures, respectively. Substantial steric clashes suggest mutually exclusive binding modes. XRCC1 has been proposed to bind damaged DNA on the opposite side of DNA to that bound by polymerase β [16]. Such binding might also be compatible with simultaneous binding by PARP-1 during the process of DNA damage recognition and repair.

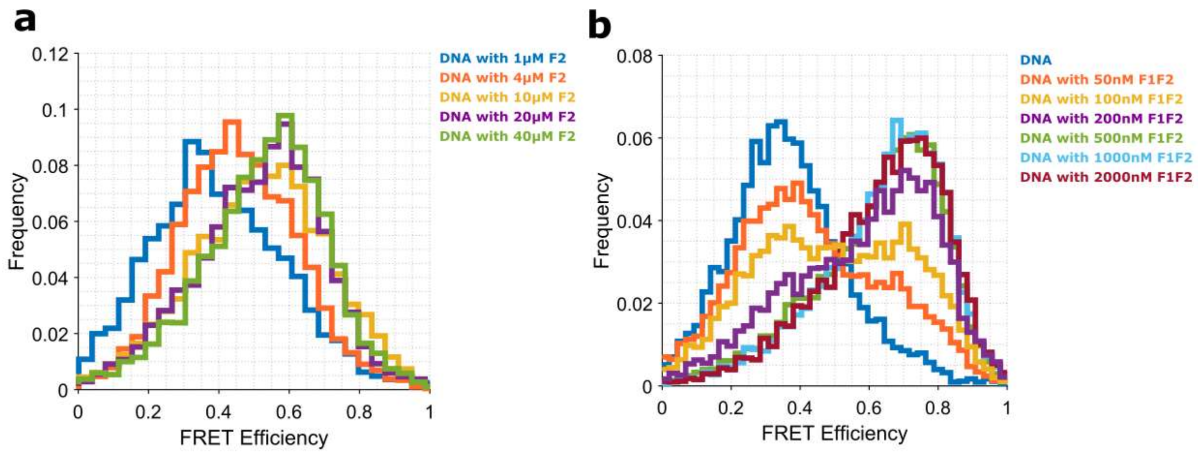

Supplementary Figure 19: Argumentation for the excess amounts of protein used in smFRET measurements on the example of DNA_{Tamra}.

a: smFRET efficiency histograms of nicked DNA in presence of 1,4,10,20 and 40µM F2. **b:** smFRET efficiency histograms of nicked DNA in presence of 0, 50, 100, 200, 500, 1000 and 2000µM F1F2. Starting from 10µM F2 (a, yellow)**,** DNA_{Tamra} bound by F2 histogram is not changing, whereas DNA_{Tamra} bound by F1F2 is reaching its maximum FRET efficiency at already 500nM (b, green). 1µM of F1F2 was used in all smFRET measurements for convenience reasons and 1µM of PARP-1 for consistency with F1F2.

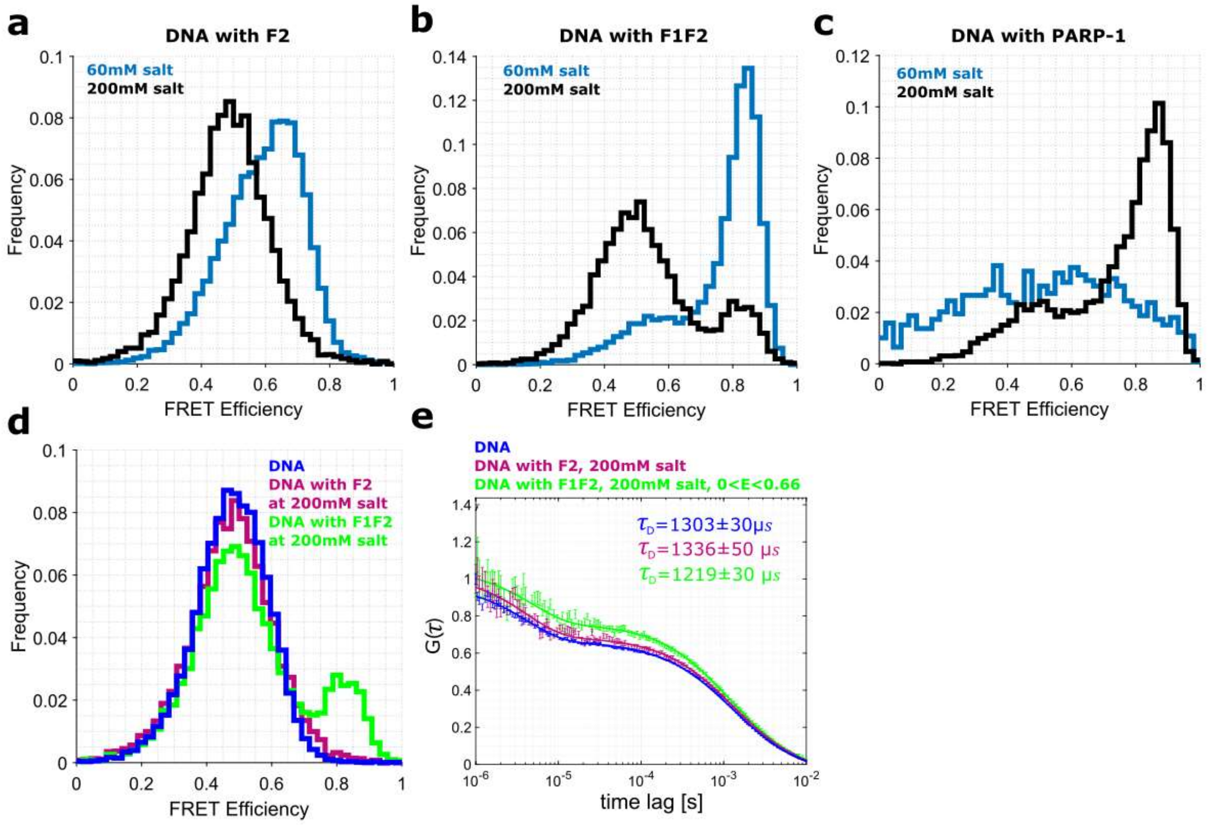

Figure S20: Salt dependent binding of F2, F1F2 and full length PARP-1 to the DNA ligand.
smFRET efficiency histograms of nicked DNA in presence of either 10µM F2 (**a**), 1µM F1F2 (**b**) or 1µM full length PARP-1 (**c**) at 60mM (blue) and 200mM (black) salt concentrations. **d:** smFRET efficiency histograms of nicked DNA (blue), DNA in presence of F2 in 200mM salt buffer (magenta, black in a), DNA in presence of F1F2 in 200mM salt (green, black in b), data are only re-plotted for clarity. **e:** Fitted FRET-FCS autocorrelation functions of DNA, DNA +F2 in 200mM salt and low FRET population (0<E<0.66) of DNA+F1F2 in 200mM salt with the same color coding as in d. The diffusion times in the top right corner of the FRET-FCS plot indicate that the three diffusion times are corresponding to free DNA; complete fit results are shown in Supp. Table S6. In c, at 60mM salt, full length PARP-1 binds nicked DNA unspecifically resulting in a broad FRET efficiency distribution (blue). In summary, optimal salt concentration for F2 and F1F2 is 60mM, whereas full length PARP-1 shows specific binding at 200mM salt concentration. The error bars show the standard error of the mean the computed molecules contributing to the same bin.

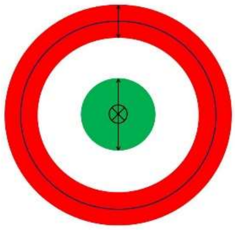

Supplementary Figure 21: Top view on a spherical cone (green) and an open spherical sector (red). Both are directed along the same axis here, indicated by the black crossed circle (center). This axis is also the mean axis of the cone, which does not need to coincide with the 3'-stem. However, we assume that the open spherical sector is directed along the 3'-axis. Its degenerate mean orientation is here shown by the black ring. The standard deviations along the spherical cone or the open spherical sector are indicated by a black double arrows.

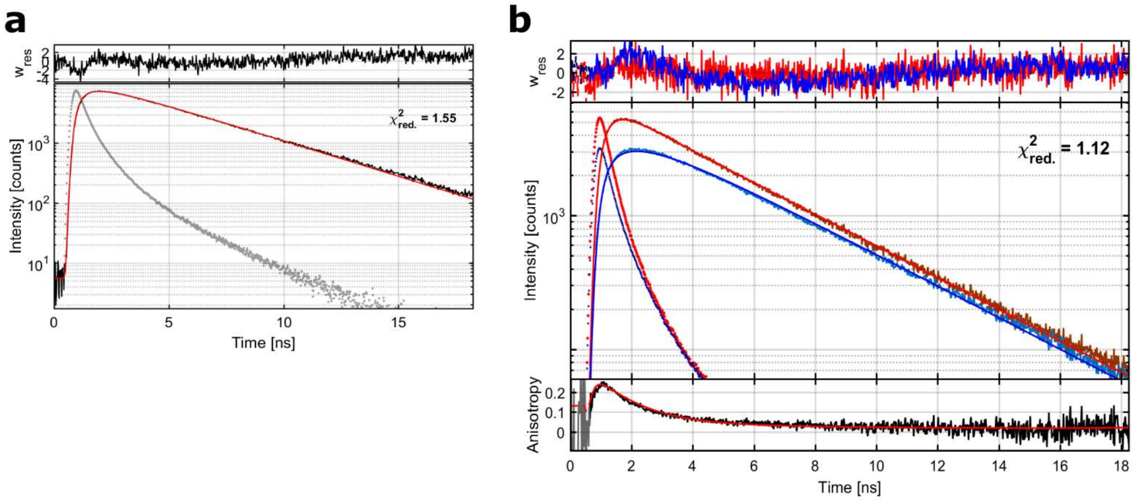

Supplementary Figure 22: Exemplary lifetime and anisotropy fits and fit results for DNA$_{Atto}$ in presence of F1F2.

Lifetime and anisotropy analysis (Supplementary methods) for DNA, DNA+F2 and DNA+F1F2 was performed to obtain the parameters (Supp. Table S4) necessary for the structural analysis (Methods). **a:** Fitted (red) donor fluorescence decay histogram (black), instrument response function (grey); the weighted residuals (w_{res}) are shown in the top panel of the fluorescence decay. The fit returned a value of $\tau_1 = 3.52n.$ **b:** Fitted donor time-resolved polarized fluorescence decays (red is the parallel channel, blue is the perpendicular channel) and the dotted decays correspond to respective IRFs; the lower panel shows the anisotropy decay curve (black) and its fit function (red); the weighted residuals (w_{res}) for both channels are shown in the top panel of the fluorescence decay. The parameters returned from the fit are $r_0 = 0.27n$, $r_\infty = 0.04ns$, $\rho_1 = 1.31$, for which the lifetime was fixed to the values given in a.

Supplementary Table 1: Results of Gaussian fitting of smFRET efficiency histograms in Supplementary Fig. S2. *A*: area fraction, *E*: mean FRET efficiency, σ: standard deviation of the Gaussian. FRET efficiencies of relevant species are highlighted according to the color code in Supplementary Fig.S2.

	A_1	E_1	σ_1	A_2	E_2	σ_2	A_3	E_3	σ_2	R^2
DNA	0.77	0.499	0.096	0.23	0.39	0.141				0.81
ligated DNA	0.75	0.484	0.092	0.25	0.39	0.126				1.74
DNA with F2	0.22	0.69	0.567	0.19	0.49	0.155	0.56	0.61	0.117	1.89
DNA with F1F2	0.44	0.86	0.039	0.30	0.58	0.144	0.25	0.77	0.056	2.78
DNA with PARP-1	0.35	0.87	0.040	0.35	0.53	0.157	0.29	0.78	0.066	1.87
DNA with PARP-1 and Eb-47	0.45	0.87	0.038	0.19	0.57	0.154	0.36	0.78	0.067	2.50
DNA with PARP-1 and olparib	0.34	0.86	0.040	0.30	0.55	0.169	0.34	0.77	0.067	2.47
DNA with PARP-1 and talazoparib	0.36	0.87	0.036	0.28	0.61	0.175	0.35	0.80	0.064	1.34
DNA with PARP-1 and niraparib	0.32	0.81	0.062	0.46	0.38	0.142	0.22	0.69	0.086	1.67
DNA with PARP-1 and rucaparib	0.39	0.84	0.48	0.27	0.51	0.179	0.34	0.75	0.075	1.27
DNA with PARP-1 and veliparib	0.40	0.86	0.38	0.26	0.56	0.154	0.33	0.78	0.063	1.72

Supplementary Table 2: fFCS fit results for DNA with F2, F1F2, PARP-1 and PARP-1 with PARPi. For each dataset two auto- (Species 1 x Species 1 and Species 2 x Species 2) and two cross-correlation functions (Species 1 x Species 2 and Species 2 x Species 1) were calculated and then fitted (Methods). From the fit, the parameters shown in the table were returned: N: mean number of molecules in the confocal volume, τ_D: diffusion time, τ: relaxation time with the respective fraction A and τ_{phot}: relaxation time due to acceptor photophysics and its respective fraction A_{phot}, y_0: offset and the weighted residuals χ^2 indicating the goodness of the fit. The blue shaded values were fixed according to previous FCS experiments (Supplementary Fig. S6) and the gray shaded value results from a global fit for the different correlations.

Data set	Correlation	N	τ_D[μs]	τ[μs]	A	τ_{phot}[μs]	A_{phot}	y_0	χ^2
DNA with F2	Sp1 x Sp1	0.036	1411	404	0.44	7.01	0.37	-0.64	3.19
	Sp1 x Sp2	0.040			0.27		0.63	-0.58	2.47
	Sp2 x Sp1	0.041			0.29		0.52	-0.50	2.19
	Sp2 x Sp2	0.016			0.20		0.59	-1.67	2.40
DNA with F1F2	Sp1 x Sp1	0.029	1587	265	0.40	7.38	0.21	-0.71	1.90
	Sp1 x Sp2	0.075			0.44		0.37	-0.40	1.28
	Sp2 x Sp1	0.075			0.44		0.37	-0.27	1.32
	Sp2 x Sp2	0.019			0.09		0.28	-1.4	1.91
DNA with PARP-1	Sp1 x Sp1	0.042	2342	273	0.43	9.58	0.22	-0.65	0.87
	Sp1 x Sp2	0.099			0.37		0.43	-0.53	1.34
	Sp2 x Sp1	0.100			0.38		0.47	-0.39	1.22
	Sp2 x Sp2	0.018			0.053		0.31	-1.88	1.44
DNA with PARP-1 and Eb-47	Sp1 x Sp1	0.042	2342	236	0.37	9.58	0.27	-0.42	0.67
	Sp1 x Sp2	0.078			0.27		0.26	-0.49	0.78
	Sp2 x Sp1	0.087			0.24		0.34	-0.44	1.00
	Sp2 x Sp2	0.021			0.04		0.22	-2.13	0.99
DNA with PARP-1 and olparib	Sp1 x Sp1	0.054	2342	241	0.48	9.58	0.23	-0.46	0.58
	Sp1 x Sp2	0.082			0.29		0.35	-0.55	0.72
	Sp2 x Sp1	0.104			0.36		0.33	-0.33	1.15
	Sp2 x Sp2	0.018			0.06		0.27	-2.34	1.07
DNA with PARP-1 and talazoparib	Sp1 x Sp1	0.017	2342	245	0.24	9.58	0.18	-2.60	0.60
	Sp1 x Sp2	0.049			0.21		0.33	-1.15	0.71
	Sp2 x Sp1	0.052			0.21		0.30	-0.95	1.09
	Sp2 x Sp2	0.018			0.054		0.23	-2.87	1.03
DNA with PARP-1 and niraparib	Sp1 x Sp1	0.065	2342	328	0.35	9.58	0.23	0.70	1.47
	Sp1 x Sp2	0.100			0.52		0.58	-0.21	0.57
	Sp2 x Sp1	0.087			0.40		0.52	-0.16	0.67
	Sp2 x Sp2	0.010			0.07		0.39	-2.35	0.72
DNA with PARP-1 and rucaparib	Sp1 x Sp1	0.030	2342	365	0.53	9.58	0.52	-1.14	1.89
	Sp1 x Sp2	0.051			0.33		0.27	-0.96	1.44
	Sp2 x Sp1	0.061			0.38		0.44	-0.80	1.14
	Sp2 x Sp2	0.015			0.06		0.34	-2.44	1.52
DNA with PARP-1 and veliparib	Sp1 x Sp1	0.045	2342	338	0.53	9.58	0.30	-0.69	1.27
	Sp1 x Sp2	0.072			0.28		0.33	-0.74	1.02
	Sp2 x Sp1	0.084			0.31		0.39	-0.57	1.28
	Sp2 x Sp2	0.019			0.10		0.25	-2.19	1.62

Supplementary Table 3: Protein Sequence of the PARP-1 fragments, PARP-1 and XRCC-1

**F1, 1-103 (11.6kDA)**
MAESSDKLYRVEYAKSGRASCKKCSESIPKDSLRMAIMVQSPMFDGKVPHWYHFSCFWKVGHSIRHPDVEVDGFSE<br>LRWDDQQKVKKTAEAGGVTGKGQDGIG
**F2, 103-214 (12.6kDA)**
GSKAEKTLGDFAAEYAKSNRSTCKGCMEKIEKGQVRLSKKMVDPEKPQLGMIDRWYHPGCFVKNREELGFRPEYSA<br>SQLKGFSLLATEDKEALKKQLPGVKSEGKRKGDEVD
**F1F2, 1-214 (24kDa)**
MAESSDKLYRVEYAKSGRASCKKCSESIPKDSLRMAIMVQSPMFDGKVPHWYHFSCFWKVGHSIRHPDVEVDGFSE<br>LRWDDQQKVKKTAEAGGVTGKGQDGIGSKAEKTLGDFAAEYAKSNRSTCKGCMEKIEKGQVRLSKKMVDPEKPQL<br>GMIDRWYHPGCFVKNREELGFRPEYSASQLKGFSLLATEDKEALKKQLPGVKSEGKRKGDEVD
**PARP-1 full length, 1-1014 (113kDa)**
MAESSDKLYRVEYAKSGRASCKKCSESIPKDSLRMAIMVQSPMFDGKVPHWYHFSCFWKVGHSIRHPDVEVDGFSE<br>LRWDDQQKVKKTAEAGGVTGKGQDGIGSKAEKTLGDFAAEYAKSNRSTCKGCMEKIEKGQVRLSKKMVDPEKPQL<br>GMIDRWYHPGCFVKNREELGFRPEYSASQLKGFSLLATEDKEALKKQLPGVKSEGKRKGDEVDGVDEVAKKKSKKEK<br>DKDSKLEKALKAQNDLIWNIKDELKKVCSTNDLKELLIFNKQQVPSGESAILDRVADGMVFGALLPCEECSGQLVFKS<br>DAYYCTGDVTAWTKCMVKTQTPNRKEWVTPKEFREISYLKKLKVKKQDRIFPPETSASVAATPPPSTASAPAAVNSSA<br>SADKPLSNMKILTLGKLSRNKDEVKAMIEKLGGKLTGTANKASLCISTKKEVEKMNKKMEEVKEANIRVVSEDFLQDV<br>SASTKSLQELFLAHILSPWGAEVKAEPVEVVAPRGKSGAALSKKSKGQVKEEGINKSEKRMKLTLKGGAAVDPDSGLE<br>HSAHVLEKGGKVFSATLGLVDIVKGTNSYYKLQLLEDDKENRYWIFRSWGRVGTVIGSNKLEQMPSKEDAIEHFMKL<br>YEEKTGNAWHSKNFTKYPKKFYPLEIDYGQDEEAVKKLTVNPGTKSKLPKPVQDLIKMIFDVESMKKAMVEYEIDLQK<br>MPLGKLSKRQIQAAYSILSEVQQAVSQGSSDSQILDLSNRFYTLIPHDFGMKKPPLLNNADSVQAKVEMLDNLLDIEV<br>AYSLLRGGSDDSSKDPIDVNYEKLKTDIKVVDRDSEEAEIIRKYVKNTHATTHNAYDLEVIDIFKIEREGECQRYKPFKQL<br>HNRRLLWHGSRTTNFAGILSQGLRIAPPEAPVTGYMFGKGIYFADMVSKSANYCHTSQGDPIGLILLGEVALGNMYE<br>LKHASHISKLPKGKHSVKGLGKTTPDPSANISLDGVDVPLGTGISSGVNDTSLLYNEYIVYDIAQVNLKYLLKLKFNFKTS<br>LW
**XRCC-1, 1-638 (70kDA)**
MGSMPEIRLRHVVSCSSQDSTHCAENLLKADTYRKWRAAKAGEKTISVVLQLEKEEQIHSVDIGNDGSAFVEVLVGSS<br>AGGAGEQDYEVLLVTSSFMSPSESRSGSNPNRVRMFGPDKLVRAAAEKRWDRVKIVCSQPYSKDSPFGLSFVRFHSP<br>PDKDEAEAPSQKVTVTKLGQFRVKEEDESANSLRPGALFFSRINKTSPVTASDPAGPSYAAATLQASSAASSASPVSRA<br>IGSTSKPQESPKGKRKLDLNQEEKKTPSKPPAQLSPSVPKRPKLPAPTRTPATAPVPARAQGAVTGKPRGEGTEPRRP<br>RAGPEELGKILQGVVVVLSGFQNPFRSELRDKALELGAKYRPDWTRDSTHLICAFANTPKYSQVLGLGGRIVRKEWVL<br>DCHRMRRRLPSRRYLMAGPGSSSEEDEASHSGGSGDEAPKLPQKQPQTKTKPTQAAGPSSPQKPPTPEETKAASPV<br>LQEDIDIEGVQSEGQDNGAEDSGDTEDELRRVAEQKEHRLPPGQEENGEDPYAGSTDENTDSEEHQEPPDLPVPELP<br>DFFQGKHFFLYGEFPGDERRKLIRYVTAFNGELEDYMSDRVQFVITAQEWDPSFEEALMDNPSLAFVRPRWIYSCNE<br>KQKLLPHQLYGVVPQAVE

Supplementary Table 4: Input parameters for calculation of accessible volumes. Atom C7 of thymidine T37 or T18 was used as attachment point for donor and acceptor, respectively. For the acceptor Alexa647, the diameter of 12Å represents a balance between the different dimensions of the dye, as it has a rather elongated structure.

		**DNA_{Atto}**		**DNA_{Tamra}**	
		**donor**	**acceptor**	**donor**	**acceptor**
dye diameter	**d [Å]**	12	12	12	12
linker length	**l [Å]**	13	13	13	18
linker width	**w [Å]**	4.5	4.5	4.5	4.5
skel. dist.	**s [Å]**	2		2	

Supplementary Table 5: Input parameters for simulation of FRET efficiencies.

		**DNA_{Atto}**	**DNA_{Tamra}**
Förster radius	**R_0 [Å]**	70	68
lifetime D	**τ_D [ns]**	3.53	2.57
rot. corr. time D	**ρ_D [ns]**	1.34	0.79
residual aniso. D	**$r_{\infty D}$**	0.042	0.153
rot. corr. time A	**ρ_A [ns]**	0.70	0.34
residual aniso. A	**$r_{\infty A}$**	0.126	0.154
number of excitations	**trajN**	50000	50000

Supplementary Table 6: FRET-FCS fit results corresponding to Fig.7 and Supp. Fig. 13: N is the average number of molecules in observation volume, τ_D is the diffusion time, τ_{phot} is a time scale characterizing acceptor blinking, A_{phot} is the fraction of the blinking and y_0 is the offset (Methods). An increased τ_{phot} is observed for DNA bound by protein datasets**.**

Data Set	N	τ_D [µs]	τ_{phot} [µs]	A_{phot}	y_0	χ^2
**DNA_{Tamra}**	0.025	1432±40	7.58	0.29	-1.36	2.23
**DNA_{Tamra}+F1**	0.041	1664±60	15.3	0.28	-0.81	1.04
**DNA_{Tamra}+F2**	0.026	1871±50	7.96	0.27	-1.67	2.74
**DNA+XRCC-1**	0.033	1502±60	4.28	0.25	-0.96	1.34
**DNA+F2**	0.040	1652±70	4.99	0.30	-0.60	1.43
**DNA+F2+XRCC-1**	0.028	1806±80	5.27	0.26	-1.25	1.04
**DNA+F1F2**	0.046	1859±20	4.67	0.30	-0.59	2.26
**DNA+F1F2+XRCC-1**	0.027	1916±60	5.53	0.24	-1.34	1.31
**DNA+PARP-1**	0.030	2422±80	4.32	0.27	-1.30	1.11
**DNA+PARP-1+XRCC1**	0.037	2780±110	4.80	0.26	-1.07	1.15
**DNA+F2+XRCC-1 (0<E<0.34)**	0.023	1741±100	6.18	0.25	-1.58	0.65
**DNA+F2+XRCC-1 (0.63<E<0.69)**	0.022	1906±90	7.55	0.19	-2.51	0.96
**DNA+F2+XRCC-1 (0.86<E<1.0)**	0.023	1965±80	6.69	0.21	-2.49	0.88
**DNA+F2 (200mM)**	0.028	1336±50	4.14	0.32	-0.87	0.69
**DNA+F1F2 (200mM, 0<E<.66)**	0.029	1219±30	4.18	0.27	-0.68	0.82

Supplementary Table 7: Correction factors applied for all datasets.

	**DNA_{AT550}**				**DNA_{Tamra}**
	**F2, F1F2**	**PARP-1, PARPi, PARP-1+XRCC1**	**F2+ XRCC1**	**F1F2+ XRCC1**	
γ	0.33	0.29	0.039	0.34	0.64
β	1.84	1.67	1.63	1.79	1.49
α	0.064	0.059	0.059	0.056	0.085
δ	0.089	0.130	0.130	0.130	0.131

Supplementary Table 8: Statistics of events in all datasets before and after isolating the FRET population.

Dataset	Number of events before filters	Number of events after filters	Acquisition time	Used in
DNA	87739	25301	3h	Fig.1c; Fig.2; Fig.6a,b,c; Fig.7a,b,c; Supp. Fig. S2b; Supp. Fig. S8a,b,c; Supp. Fig. S9; Supp. Fig. S10; Supp. Fig.S11; Supp. Fig. S12a,b,c; Supp. Fig. S13a,b; Supp. Fig.S20d,e
ligated DNA	66844	18666	2h	Fig.1c, Supp. Fig. S2a;
DNA with PARP-1 (2021)	154399	20278	6.5h	Fig.1c; Fig.2; Fig.5c; Fig.6a,b,c; Supp. Fig. S2e; Supp. Fig. S8a,b,c; Supp. Fig. S9; Supp. Fig. S9
DNA with F2	151208	40504	7h	Fig.2; Fig.5a; Fig.7b,d; Supp. Fig. S2c; Supp. Fig. S8a,b,c; Supp. Fig. S9; Supp. Fig.S19a;
DNA with F1F2	257765	61990	9h	Fig.2: Fig.5b; Supp. Fig. S2d; Supp. Fig. S8a,b,c; Supp. Fig. S9; Supp. Fig.S15b,d; Supp. Fig. S22a,b;
DNA with PARP-1 and Eb-47	85456	11022	8h	Fig.6a; Supp. Fig. S2f;
DNA with PARP-1 and olparib	165528	9960	3.5h	Fig.6b; Supp. Fig. S2g;
DNA with PARP-1 and niraparib	156969	13225	5h	Fig.6c; Supp. Fig. S2i; Supp. Fig. S10;
DNA with XRCC-1	58803	7862	1.5h	Fig. 7a; Supp. Fig. S16a;
DNA with F2 and 1μM XRCC-1	94605	12529	3h	Fig. 7b,d; Supp. Fig. S16b; Supp. Fig. S17a,b;
DNA_{Tamra}	150099	14466	5h	Supp. Fig. S3a;
DNA_{Tamra} with F2	225272	25501	8h	Supp. Fig. S3a;
DNA_{Tamra} with F1F2	168367	15039	4h	Sup. Fig. S3a;
DNA_{Tamra} with F1	8396	1511	0.5h	Supp. Fig S3a,b;
DNA with PARP-1 and talazoparib	138156	6073	7.5h	Supp. Fig. S2h; Supp. Fig. S12a;
DNA with PARP-1 and veliparib	72434	7997	4.5h	Supp. Fig. S2k; Supp. Fig. S12b;
DNA with PARP-1 and rucaparib	253071	14861	11h	Supp. Fig. S2j; Supp. Fig. S12c;
DNA with PARP-1 (2022)	197862	16573	6h	Supp. Fig. S9a,b,c; Supp. Fig. S15a,c
DNA with EB-47	59502	9643	1.5h	Supp. Fig. S13a;
DNA with olparib	29584	3382	1h	Supp. Fig. S13a;
DNA with talazoparib	55819	12799	1.5h	Supp. Fig. S13a,b;
DNA with niraparib	70455	8409	1.5h	Fig. 6c; Supp. Fig. S11; Supp. Fig. S13a,b;
DNA with veliparib	57449	4989	1h	Supp. Fig. S13a;
DNA with rucaparib	19604	4814	1h	Supp. Fig. S9c; Supp. Fig. S13a,b;
DNA with PARP-1 and 1μM XRCC-1	13332	1961	0.5h	Supp. Fig. S15a,c;
DNA with F1F2 and 1μM XRCC-1	90734	9515	2.5h	Supp. Fig. S15b,d;
DNA_{Tamra} with 1μM F2	16005	4433	0.5h	Supp. Fig. S19a;
DNA_{Tamra} with 4μM F2	38454	5091	0.5h	Supp. Fig. S19a;
DNA_{Tamra} with 10μM F2	19460	5162	0.5h	Supp. Fig S3a,b; Supp. Fig. S19a;
DNA_{Tamra} with 20μM F2	22404	6703	0.5h	Supp. Fig. S19a;
DNA_{Tamra} with 40μM F2	23894	6710	0.5h	Supp. Fig. S19a;
DNA_{Tamra}	55238	6762	1h	Supp. Fig S3a,b; Supp. Fig. S19b;
DNA_{Tamra} with 50nM F1F2	50853	5797	1h	Supp. Fig. S19b;
DNA_{Tamra} with 100nM F1F2	54803	6129	1h	Supp. Fig. S19b;
DNA_{Tamra} with 200nM F1F2	39712	4258	1h	Supp. Fig. S19b;
DNA_{Tamra} with 500nM F1F2	53930	6070	1h	Supp. Fig. S19b;
DNA_{Tamra} with 1000nM F1F2	46324	5091	1h	Supp. Fig. S19b;
DNA_{Tamra} with 2000nM F1F2	41727	4996	1h	Supp. Fig. S19b;
DNA with F2, 200mM salt	54367	6237	1h	Supp. Fig. S20a,d,e;
DNA with F1F2, 200mM salt	62611	7577	1h	Supp. Fig. S20b,d,e;
DNA with PARP-1, 60mM salt	33601	1404	0.5h	Supp. Fig. S20c;

Supplementary references

1. Eustermann, S. *et al.* Structural Basis of Detection and Signaling of DNA Single-Strand Breaks by Human PARP-1. *Mol. Cell* **60**, 742–754 (2015).
2. Taniguchi, M., Du, H. & Lindsey, J. S. PhotochemCAD 3: Diverse Modules for Photophysical Calculations with Multiple Spectral Databases. *Photochem. Photobiol.* **94**, 277–289 (2018).
3. Kallis, E. Quantitative single-molecule FRET and its application in experiments on DNA damage recognition by PARP-1. *PhD disseration* (2020).
4. Hellenkamp, B. *et al.* Precision and accuracy of single-molecule FRET measurements-a multi-laboratory benchmark study. *Nat. Methods* **15**, 669–676 (2018).
5. Stennett, E. M. S., Ciuba, M. A., Lin, S. & Levitus, M. Demystifying PIFE: The Photophysics Behind the Protein-Induced Fluorescence Enhancement Phenomenon in Cy3. *J. Phys. Chem. Lett.* **6**, 1819–23 (2015).
6. Kalinin, S., Valeri, A., Antonik, M., Felekyan, S. & Seidel, C. A. M. Detection of Structural Dynamics by FRET: A Photon Distribution and Fluorescence Lifetime Analysis of Systems with Multiple States. *J. Phys. Chem. B* **114**, 7983–7995 (2010).
7. Torella, J. P., Holden, S. J., Santoso, Y., Hohlbein, J. & Kapanidis, A. N. Identifying Molecular Dynamics in Single-Molecule FRET Experiments with Burst Variance Analysis. *Biophys. J.* **100**, 1568–1577 (2011).
8. Gopich, I. V. & Szabo, A. Single-molecule FRET with diffusion and conformational dynamics. *J. Phys. Chem. B* **111**, 12925–12932 (2007).
9. Schwille, P., Meyer-Almes, F. J. & Rigler, R. Dual-color fluorescence cross-correlation spectroscopy for multicomponent diffusional analysis in solution. *Biophys. J.* **72**, 1878–1886 (1997).
10. Torres, T. & Levitus, M. Measuring conformational dynamics: A new FCS-FRET approach. *J. Phys. Chem. B* **111**, 7392–7400 (2007).
11. Ries, J. & Schwille, P. Fluorescence correlation spectroscopy. *BioEssays* **34**, 361–368 (2012).
12. Langelier, M.-F., Planck, J. L., Roy, S. & Pascal, J. M. Crystal Structures of Poly(ADP-ribose) Polymerase-1 (PARP-1) Zinc Fingers Bound to DNA. *J. Biol. Chem.* **286**, 10690–10701 (2011).
13. Eustermann, S. *et al.* The DNA-binding domain of human PARP-1 interacts with DNA single-strand breaks as a monomer through its second zinc finger. *J. Mol. Biol.* **407**, 149–170 (2011).
14. Tsutakawa, S. E. *et al.* Human flap endonuclease structures, DNA double-base flipping, and a unified understanding of the FEN1 superfamily. *Cell* **145**, 198–211 (2011).
15. Sawaya, M. R., Prasad, R., Wilson, S. H., Kraut, J. & Pelletier, H. Crystal Structures of Human DNA Polymerase β Complexed with Gapped and Nicked DNA: Evidence for an Induced Fit Mechanism ,. *Biochemistry* **36**, 11205–11215 (1997).
16. Marintchev, A. *et al.* Solution structure of the single-strand break repair protein XRCC1 N-terminal domain. *Nat. Struct. Biol.* **6**, 884–893 (1999).
